# Vein Grafting in Free Flap Surgery for Head and Neck Oncology: Necessity, Innovation, or Complication?

**DOI:** 10.1097/SCS.0000000000012594

**Published:** 2026-03-24

**Authors:** Salvatore Battaglia, Salvatore Crimi, Matteo Grimaldi, Dario Ortoleva, Cesare D’Amico, Marco Cicciù, Alberto Bianchi

**Affiliations:** * Department of General Surgery, Section of Maxillo-Facial Surgery of Polyclinic “G. Rodolico—San Marco” University Hospital, University of Catania; † Department of General Surgery and Surgical-Medical Specialties, School of Dentistry, University of Catania, Catania; ‡ Department of Biomedical and Dental Sciences, Morphologic and Functional Images, University of Messina, Messina, Italy

**Keywords:** Head and neck oncology, microvascular free flap, vein grafting

## Abstract

**Objective:**

Vein grafting may provide a valuable option in microvascular free flap reconstruction for head and neck cancer when direct anastomosis is not feasible. This study assesses indications and outcomes, reappraises prevailing assumptions, and delineates clinical contexts in which the technique could enhance reconstructive reliability and success.

**Methods:**

This retrospective study evaluates the use of vein grafts in microvascular free flap reconstruction for head and neck malignancies. Among 51 consecutive patients treated at S. Marco Hospital, University of Catania (2021–2024), 11 underwent vein grafting. In 4 cases, grafting was adopted as a secondary measure following anastomotic failure or excessive vessel distance that precluded direct anastomosis; in 7, it was planned from the outset due to anticipated vessel misalignment or insufficient pedicle length. The external jugular vein served as the graft conduit in all cases.

**Results:**

Among 11 patients, 5 underwent radial forearm flap, 2 fibular flap, 2 anterolateral thigh (ALT), and 2 latissimus dorsi flap. Four patients underwent radiotherapy, whereas 2 are still awaiting evaluation. Only 1 case resulted in flap necrosis, requiring surgical revision. Complications were minimal, with 1 case of wound dehiscence. No cases of venous thrombosis, arterial insufficiency, or total flap loss occurred beyond the single necrosis case. The overall flap survival rate was 91%.

**Conclusions:**

Vein grafting is a safe, effective adjunct for head and neck free flap reconstruction, particularly when pedicle-recipient mismatch or thrombosis precludes direct anastomosis, and can improve outcomes in complex cases.

Microvascular free flap reconstruction has become a cornerstone in the surgical management of head and neck cancers, offering the dual benefits of restoring both function and esthetics following extensive tumor resections.^1^

Since Crowell and Yasargil^2^ initially introduced microvascular autografting techniques, concerns regarding the dependability of free tissue transfer utilizing vein grafts have emerged.

The success rates of these procedures have markedly improved over the decades, with current literature reporting survival rates ranging from 91% to 95%.^1,3–5^ However, certain clinical scenarios present challenges to direct vessel anastomosis, such as prior surgeries, radiation therapy, or anatomic variations that compromise the availability or quality of recipient vessels. In these situations, interposition vein grafting emerges as a viable solution to bridge vascular gaps and facilitate successful free flap transfers.^6^ Despite its utility, vein grafting is often perceived as a last-resort technique, primarily due to concerns about increased risks of flap compromise and loss. Several studies have highlighted a significant occurrence of thrombotic complications and flap failure associated with the use of vein grafts.^6–9^ Therefore, reevaluating the role of vein grafting is crucial to optimize reconstructive outcomes in head and neck oncology. This article aims to critically evaluate the role of vein grafting in microvascular free flap reconstruction for head and neck cancer patients, examining surgical indications, clinical outcomes, and potential complications. By challenging conventional perceptions and highlighting scenarios in which vein grafting could redefine reconstructive success, we seek to provide an updated, evidence-based perspective on this underutilized technique.

## METHODS

This is a retrospective study evaluating the use of vein grafts in microvascular free flap reconstruction for head and neck oncology. A total of 11 patients out of 51 who underwent reconstructive surgery between 2021 and 2024 at S. Marco Hospital, University of Catania, were included in this analysis. Seven patients were female, and 4 men, and the average age was 62 years. Of the 11 patients in the study, 5 had mandibular involvement, 2 had oral cavity involvement, 1 had maxillary involvement, and 3 had buccal mucosa involvement. Half of the cohort smoked cigarettes; 5 patients had cardiovascular disease, 4 had diabetes, whereas the remaining part of the group had no comorbidities that could influence the success of the free flap. All patients underwent a preoperative interview, during which the surgical procedure was explained in detail, including the potential need for a vein graft, and the possible risks and complications intraoperative, perioperative, and postoperative.

**FIGURE 1:**
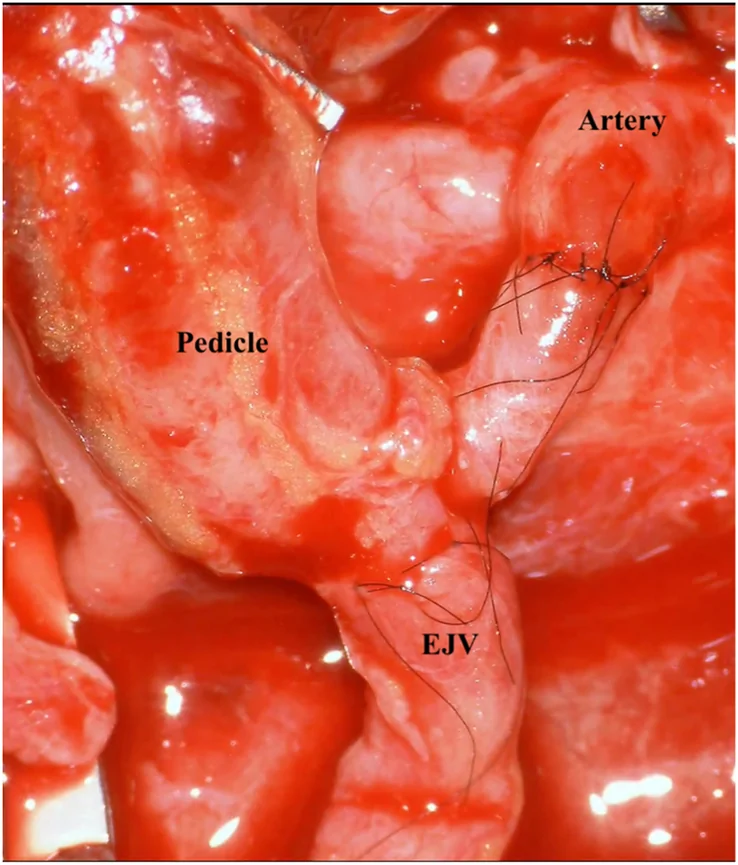
Image of the external jugular vein serving as the sole graft in the surgical site. EJV.

Vein grafts were harvested exclusively from the surgical site in the neck, with the external jugular vein serving as the sole graft source for all 11 patients (Fig. 1). The grafts were then used to establish vascular continuity between the free flap and recipient vessels, facilitating successful microvascular anastomosis. All surgeries were performed by a team of experienced surgeons specializing in head and neck reconstruction, with close monitoring of the patients during the postoperative period for any signs of graft failure or complications.

## RESULTS

A total of 11 patients underwent microvascular free flap reconstruction with vein graft interposition. Among them, 10 patients had been diagnosed with squamous cell carcinoma of the oral cavity, whereas 1 patient had verrucous carcinoma. No patient was subjected to radiotherapy treatment before the surgical procedure. Of the 11 reconstructions performed, 4 patients received radial forearm free flaps, 2 received fibular flaps, 2 received latissimus dorsi flaps, and 2 received anterolateral thigh (ALT) flaps. Among the 11 patients, 4 required vein grafting as a secondary procedure to address complications such as previous anastomotic failure or an excessive distance between the recipient and donor veins, which made direct anastomosis unfeasible. In the remaining 7 cases, vein grafting was selected as the primary strategy from the outset, following intraoperative assessments that predicted unfavorable vessel positioning or insufficient pedicle length. In 7 cases, an end-to-side anastomosis was performed on the internal jugular vein, in 2 cases an end-to-end anastomosis on the lingual vein, and in the remaining 2 cases, an end-to-end anastomosis was performed on the superior thyroid vein (Table 1, Supplemental Digital Content 1, http://links.lww.com/SCS/J251).

All patients followed the same antithrombotic protocol consisting of 6000 IU of heparin administered subcutaneously 6 hours after surgery and, from noon on the first postoperative day, a mix of 100 mg of cardioaspirin at 12 AM and 4000 IU of heparin every evening at 6 PM once daily, By reason of the necessity to intervene, preventing ischemic episodes, on both platelet aggregation and the coagulation cascade. The average hospital stay after surgery was 12 days. All patients spent about 72 hours in the intensive care unit for postoperative monitoring. Approximately half of the patients required blood transfusions during the perioperative period. The Hb value considered as the parameter below which it was necessary to subject patients to a blood transfusion was 9 g/dl. Postoperative complications were minimal; only 1 patient experienced wound dehiscence. No cases of venous thrombosis or arterial insufficiency were observed. We observed only 1 case of free flap necrosis; unfortunately, the patient died during hospitalization. All other patients demonstrated adequate flap perfusion and successful integration during the follow-up period.

**FIGURE 2:**
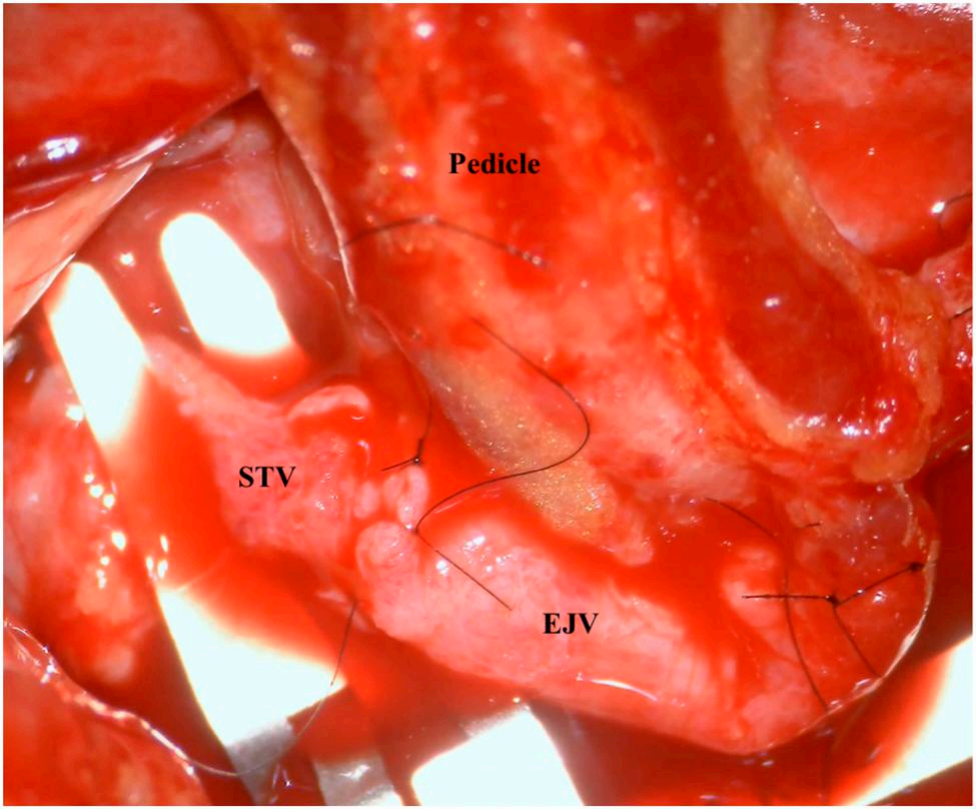
Image of the external jugular vein serving as the sole graft in the surgical site. Vein graft.

The overall flap survival rate was 91%, with only 1 case of flap necrosis requiring surgical revision (Fig. 2).

## DISCUSSION

In microvascular free flap reconstruction for head and neck oncology,^10,11^ intraoperative challenges, such as unanticipated discrepancies in vessel length, can compromise the success of the procedure. These discrepancies often arise due to individual variations in tissue anatomy, making preoperative assessments of pedicle length occasionally inaccurate. In such scenarios, the use of vein grafts becomes an optimal solution to bridge the gap between donor and recipient vessels.

A comprehensive study analyzing over 3000 free flap reconstructions found that while vein grafts were associated with higher odds of flap compromise and flap loss, an overall success rate of 93.4% was still achieved in these more complex cases.^10^ Furthermore, the challenges inherent in reconstructing recurrent head and neck cancers—which often involve scarred and irradiated tissues—underscore the importance of meticulous surgical planning and technique. Studies have shown that free flap reconstructions in recurrent cases have higher failure rates than in primary cases, emphasizing the need for tailored approaches in these complex scenarios.^3^ Despite its potential benefits, autologous vein grafting remains underutilized in microvascular free flap reconstruction for head and neck cancer. A retrospective study spanning a decade reported that vein grafts were used in only 7.4% of microvascular reconstruction cases, predominantly in scenarios involving prior surgeries, radiation therapy, or tumor recurrence.^10^ This limited application may stem from concerns about increased technical complexity and potential postoperative complications. However, evidence indicates that, with meticulous patient selection and surgical technique, the success rates of vein grafting are comparable to those of direct anastomosis.^3^

Vein grafting serves as an effective strategy to extend the vascular pedicle. This technique not only facilitates the necessary vascular continuity but also minimizes the risk of complications associated with excessive anastomotic tension, such as thrombosis or flap failure. By harvesting vein grafts from the same surgical site, surgeons can efficiently increase pedicle length without subjecting the patient to additional donor site morbidity.

The decision to use vein grafts is often made intraoperatively, underscoring the importance of surgical adaptability in response to real-time anatomic findings. This approach allows for immediate resolution of unforeseen challenges, optimizing reconstructive outcomes. Studies have demonstrated that, with meticulous surgical technique and patient selection, the success rates of vein grafting are comparable to those of direct anastomosis, reinforcing its reliability as a reconstructive option.^3^ Moreover, different studies have demonstrated that presurgical planning of vein grafts is associated with a higher rate of success.^12,13^

The efficacy of vein grafts in microvascular free flap reconstruction is underpinned by fundamental hemodynamic principles, notably Poiseuille law.^14^ This law articulates that blood flow (Q) through a vessel is directly proportional to the fourth power of the vessel’s radius (r) and the pressure gradient (ΔP), whereas being inversely proportional to the vessel’s length (L) and blood viscosity (η):


$$Q=\frac{\pi \cdot r^{4}\cdot \Delta P}{8\cdot L\cdot \eta }$$

However, in practical applications, both blood viscosity (η) and π are constants, while the pressure gradient (ΔP) is relatively small, typically around 1 to 2 mm Hg. As a result, the formula simplifies to:


$$Q\approx \frac{r^{4}}{8L}$$

This simplified equation highlights that vessel radius (r) has the most significant impact on blood flow, as it is raised to the fourth power. Poiseuille law elucidates that even minor adjustments in vessel radius can substantially influence blood flow rates (Table 2, Supplemental Digital Content 1, http://links.lww.com/SCS/J251).

Therefore, selecting a vein graft with an appropriate diameter is crucial to maintain optimal hemodynamic conditions and reduce the risk of thrombosis.^15–17^ In addition, while longer grafts might theoretically increase resistance, studies have indicated that the length of venous autografts does not significantly affect patency in microvascular anastomoses.^18–20^ Thanks to Poiseuille law, the use of vein grafts becomes a viable and effective strategy in microvascular free flap reconstruction whenever necessary. By selecting a vein graft with a larger diameter than the recipient veins typically used for anastomosis, the blood flow is significantly increased due to the fourth-power relationship between vessel radius and flow. This results in improved perfusion of the flap, optimizing oxygen and nutrient delivery to the transplanted tissue. One possible criticism that could be raised regarding the use of a vein graft is the size difference between the pedicle vein and the graft, which could make the anastomosis more difficult. However, this potential limitation can be easily managed by distributing it circumferentially. Moreover, the increased vascular diameter reduces the risk of turbulence and thrombosis, contributing to higher graft patency rates and better overall flap survival. This hemodynamic advantage further justifies the intraoperative decision to use vein grafts when direct anastomosis is either unfeasible or would result in excessive tension. By leveraging these physiological principles, vein grafting not only resolves intraoperative anatomic challenges but also actively contributes to the success of microvascular reconstruction by maximizing perfusion and ensuring long-term flap viability.

## CONCLUSION

We acknowledge the limited statistical power of the clinical cases discussed, given the small number of patients considered and the absence of a control group. However, the use of vein grafting in free flap microsurgery of the head and neck region should not be considered a preoperative method. Instead, it represents a salvage surgical technique, not yet described in the literature, whose use can only be determined intraoperatively. Therefore, the number of patients involved can only increase if, intraoperatively, it becomes necessary to resort to the aforementioned technique. Nevertheless, vein grafting represents a valuable and reliable solution in cases where direct anastomosis is not feasible. By extending the vascular pedicle and optimizing blood flow, vein grafts help prevent excessive anastomotic tension, reducing the risk of thrombosis and flap failure.

Several studies have demonstrated that, when performed with proper surgical technique, vein grafting does not compromise overall flap survival rates and can, in fact, improve outcomes in challenging cases.^20,21^

To scientifically accept this surgical technique as a valuable option in head and neck microvascular reconstruction, given its ability to overcome intraoperative anatomic limitations, further studies and comparisons with other centers are necessary.

## Supplementary Material
