# Supplementary material for: Vein Grafting in Free Flap Surgery for Head and Neck Oncology: Necessity, Innovation, or Complication?
Source: J Craniofac Surg. 2026 Mar 24;37(9):2262–5. doi: 10.1097/SCS.0000000000012594 (PMC13552566; doi:10.1097/SCS.0000000000012594)
Supplement: Supplementary file 1 [file scs-37-2262-s001.docx]

*Tab 1. Summary of Results*

*Tab 2. Rappresentation of key parameters and their effects according to Poiseuille's law*
